# Deep brain stimulation and revising the Mental Health Act: the case for intervention-specific safeguards

**DOI:** 10.1192/bjp.2018.200

**Published:** 2019-03

**Authors:** Jonathan Pugh, Tipu Aziz, Jonathan Herring, Julian Savulescu

**Affiliations:** 1 Research Fellow in Applied Moral Philosophy, The Oxford Uehiro Centre for Practical Ethics, University of Oxford, UK; 2 Professor of Neurosurgery, The Oxford Uehiro Centre for Practical Ethics, University of Oxford, UK; 3 Professor of Law, Faculty of Law, University of Oxford, UK; 4 Uehiro Chair in Practical Ethics, Oxford Functional Neurosurgery, University of Oxford, UK

**Keywords:** Mental Health Act, deep brain stimulation, mental health law, medical ethics, mental capacity

## Abstract

Under the current Mental Health Act of England and Wales, it is lawful to perform deep brain stimulation in the absence of consent and independent approval. We argue against the Care Quality Commission's preferred strategy of addressing this problematic issue, and offer recommendations for deep brain stimulation-specific provisions in a revised Mental Health Act.

**Declaration of interest:**

T.A. is a paid consultant for Boston Scientific, Medtronic and St. Jude Medical. He has received honoraria from Abbott, Boston and Medtronics and served as consultant to all three.

The UK government is currently reviewing the Mental Health Act (1983) of England and Wales (MHA). Here, we address a significant gap in the MHA, namely its lack of safeguards for the use of deep brain stimulation (DBS) in the treatment of psychiatric disorders. We begin with an overview of DBS in this context, before explaining how it could be lawful to perform DBS in the absence of consent under the current MHA. We then consider the Care Quality Commission's (CQC) preferred strategy of addressing this by bringing DBS within the scope of existing protections governing the use of neurosurgery for mental disorder (NMD). We raise two problems with this strategy: (a) the restrictions on lawful urgent uses of NMD are too permissive for DBS, and bringing DBS under these restrictions would introduce an arbitrary inconsistency into the MHA and (b) the MHA's certification test for capacity is not sufficiently sensitive to autonomy-based concerns in psychiatric DBS. We conclude that DBS should be treated separately from NMD in the revised MHA, with recommendations for DBS-specific provisions that would address these problems.

## DBS

Following its success in the treatment of movement disorders,^1^ DBS is being increasingly considered as an investigational therapy for patients with treatment-refractory psychiatric conditions.^2^ Although there is little consensus on its precise mechanisms of action, DBS broadly aims to achieve a therapeutic effect by delivering electrical stimulation to areas of the brain that are understood to underlie a particular pathology. To receive stimulation, patients must undergo a procedure in which electrodes are implanted into the targeted brain area and then connected to a subcutaneously implanted impulse generator. This hardware can then deliver electronic stimulation that can be titrated to the needs of the particular patient. Insofar as its effects are stimulation-dependent, DBS is reversible; stimulation can be ceased and the hardware can be explanted.^3^

Like any neurosurgical procedure, DBS is associated with perioperative risks, as well as risks associated with the long-term implantation of the device.^4^ Stimulation has also been associated with adverse cognitive, behavioural, psychiatric and psychosocial effects.^4^ In particular, the ethical discussion concerning DBS has focused on its association with adverse psychosocial effects on the patient's self-conception.^5^ There is emerging data of such effects arising in psychiatric applications of DBS;^6^ these effects may also be complicated in this context by the fact that the targeted conditions can be egosyntonic.^7^ Despite these risks, and the lack of a strong evidence base for DBS in psychiatry, there is considerable optimism regarding DBS as a last-resort intervention for some patients with treatment-refractory illness.

## DBS, NMD and the Mental Health Act

In a number of jurisdictions, mental health law permits the non-consensual medical treatment of individuals with a mental disorder, for that mental disorder.^8^ However, among those jurisdictions that permit such non-consensual treatment, some also specifically regulate certain methods of treatment. According to a report from the European Commission, NMD (or so-called ‘psychosurgery’) is specifically regulated in the European Union by Denmark, Ireland, Portugal, Germany and the UK.^9^ It is also subject to specific regulations in all Australian states, as well as some (although not all) US states and Canadian provinces or territories.^10^^–^^21^ However, there is little uniformity in these legislative instruments, either with regard to the definition of NMD (or psychosurgery) they employ, or the manner in which it is regulated.

Under (section 57 of) the MHA, surgical operations that destroy brain tissue or its functioning are only lawful in non-urgent circumstances if:

(a) the patient has been certified as both capable of understanding the nature, purpose and likely effects of the treatment, and having consented to it.
  and
(b) the treatment is deemed appropriate by an independent registered medical practitioner.

Notably though, these safeguards around consent and independent approval do not apply in urgent circumstances delineated in section 62.

Moreover, the CQC has stated that DBS does not fall within the scope of these protections.^22^ Therefore, it could technically be lawful to perform DBS in the absence of consent under the MHA, and (for the first three months of treatment) in the absence of independent approval. This is ethically unacceptable, given the experimental status of the intervention, and the significant risks associated with it.

Furthermore, other jurisdictions have passed legislation to address similar gaps in their mental health law, including Scotland^23^ and various states in the USA, Canada and Australia.^10^^–^^19^ Such legislation has typically served to bring DBS within the scope of the protections governing NMD under the jurisdiction's mental health legislation. The CQC have advocated this strategy for the MHA.^22^ Some states have even legally defined DBS as a form of NMD or ‘psychosurgery’.^10^^–^^14^

Of course, in such states the protections governing NMD may vary considerably across jurisdictions. At one extreme, section 83 of the New South Wales Mental Health Act simply prohibits all forms of ‘psychosurgery’ (which is defined in such a way as to seemingly incorporate DBS)^11^ unless the procedure is used to treat the following conditions set out in (section 10 of) the Mental Health Act Regulations: (a) Parkinson's disease; (b) Gilles de la Tourette syndrome; (c) Chronic tic disorder; (d) Tremor; or (e) Dystonia.^24^ On the other hand, in Scotland, NMD may lawfully be performed on non-resisting patients who lack capacity.^25^ The protections governing NMD in the MHA, requiring both consent and independent approval, represent something of a middle ground that has been adopted in a number of other jurisdictions.

In contrast to this strategy of subjecting NMD and DBS to the same set of regulations, the Australian state of Queensland has recently adopted an alternative strategy, by including a specific section for non-ablative neurosurgical procedures in its 2017 Mental Health Act.^15^ This section permits the consensual use of DBS following tribunal approval, despite the fact that the act prohibits the use of ablative NMD.

Contrary to the Queensland Act, other jurisdictions that subject NMD and DBS to the same set of regulations do permit NMD under certain conditions. This is in keeping with the UK Royal College of Psychiatrists’ recommendation that NMD ‘may reasonably be considered’ for ‘carefully selected patients’.^26^ We shall not address the question of whether mental health legislation should prohibit NMD. However, we shall now argue that a revised MHA should follow the Queensland Act in distinguishing NMD from DBS for the purposes of mental health legislation.

One might defend this strategy by claiming that DBS is, unlike NMD, reversible and therefore poses less post-operative risk. We shall not adopt this strategy here. In addition to disputes about the extent of DBS' reversibility,^27^ it is not clear that reversibility alone can justify subjecting DBS to weaker legal protections than NMD. Even if we assume that DBS is less risky than NMD, it might be claimed that they are both risky enough to justify safeguards of a similar strength. This thought, in conjunction with the small evidence base for both interventions, perhaps partially explains the legislative trend towards subjecting DBS to the same legal provisions as NMD.

We believe that the risks of DBS justify placing strong safeguards on its use. However, we explicitly reject the claim that DBS should be subject to the same protections as NMD, and much less that we should define DBS as a form of NMD. As we shall explore below, there are legislation-specific reasons not to subject DBS to the regulations governing NMD in the MHA. Going forward, a further reason not to define DBS as a form of NMD is that the evidence base of the former is likely to significantly develop. DBS is a rapidly advancing technology that does not face many of the scientific and ethical obstacles facing the study of NMD. Thus, to legally define DBS as a form of NMD may serve to raise unnecessary obstacles to changing the protections governing DBS in the future, if evidence justifies doing so.

## Two problems with bringing DBS under existing regulations governing NMD in the MHA

### The restrictions on lawful urgent uses of NMD are too permissive for DBS and would introduce arbitrary inconsistency

Although we have explained that the MHA regulates certain treatments (including NMD), these regulations can be overridden in emergency situations. Under section 62, any treatment can be used without consent or independent approval if it is immediately necessary to save the patient's life. Moreover, reversible treatments can also be used without consent or independent approval if it is immediately necessary to prevent a serious deterioration of the patient's condition, even if the treatment is hazardous. Finally, if a treatment is neither irreversible or hazardous, it may be used without consent or independent approval if it is immediately necessary to alleviate serious suffering, or if it is the minimum interference necessary to prevent the patient from behaving violently or being a danger to himself or to others.^28^

As a hazardous and irreversible procedure, NMD could only be performed non-consensually (and without independent approval) if it were immediately necessary to save the recipient's life. That said, it is difficult to imagine circumstances in which it would be feasible to carry out such an extensive procedure in such urgent circumstances.

However, if DBS were simply subject to the same set of regulations as NMD, DBS could plausibly be used without consent or independent approval for any of the emergency situations outlined above. Insofar as DBS is reversible, it could lawfully be used to prevent a serious deterioration of the patient's condition. Since DBS requires an intracranial procedure to implant the device, it might plausibly be suggested that the intervention poses a significant physical hazard to the patient. If so, this would preclude the use of DBS for the final two urgent uses outlined in the first paragraph of this section. However, if a patient had already undergone this hazardous procedure to implant the device, and previously undergone stimulation without serious adverse physical complications, then it could plausibly be lawful to non-consensually initiate stimulation for these purposes. Such stimulation would be reversible, and there would be little evidence to suggest that initiating stimulation would pose a significant physical hazard to the patient.

This is problematic for two reasons. First, professional guidelines have stated unequivocally that DBS should not be used for ‘political, law enforcement or social purposes’.^29^ Yet, recall that the MHA permits the non-consensual use of non-hazardous, reversible (section 57) treatments for the purpose of preventing the patient from ‘behaving violently or being a danger to himself or to others’. If DBS were to be simply brought within the scope of the (section 57) regulations governing NMD, this would leave the door open to the widely condemned possibility of using DBS for social control.

A further problem arises when we consider the above in conjunction with how electroconvulsive therapy (ECT) is regulated under the MHA. The section 58A safeguards for ECT are less restrictive than the safeguards for NMD: they permit ECT for patients who lack capacity, and ECT for capable consenting patients in the absence of independent approval. These weaker safeguards are partly a reflection of ECT's strong evidence base, at least in the treatment of major depression.^30^ However, under section 62 the safeguards governing ECT may not be lawfully overruled to alleviate serious suffering, or to prevent the patient from behaving violently or being a danger to himself or to others.

This latter feature is consistent with the MHA's treatment of NMD; NMD is subjected to stricter safeguards than ECT for both non-urgent and urgent uses. However, if DBS were to simply be brought under the exact same set of regulations as NMD, this would introduce an arbitrary inconsistency into the MHA's treatment of DBS and ECT: DBS would be subject to stricter restriction than ECT for non-urgent uses, but weaker restrictions for urgent uses.

#### Recommendation 1

To avoid the use of DBS for social purposes, and to avoid arbitrary inconsistency in the MHA's treatment of DBS and ECT, the safeguards for DBS should only not apply for emergency treatment that is immediately necessary to save life or to prevent a serious deterioration of the patient's condition.

### The MHA test for capacity is not sufficiently sensitive to autonomy-based concerns in psychiatric DBS

Under the MHA, non-urgent NMD may only be performed on a consenting patient who has been certified to be capable of ‘…understanding the nature, purpose and likely effects of the treatment in question’. Although this certification test of capacity may be sufficient for NMD, it is not sufficiently sensitive to autonomy-based concerns regarding psychiatric DBS.

As we mentioned above, DBS has been associated with adverse effects on the patient's self-conception. Notably, although it is difficult to make likewise comparisons of the relevant effects in DBS and NMD, a review published in this journal suggests that there is little evidence associating NMD with adverse effects on personality.^31^ In any case, in addition to implications for the patient's quality of life, ethicists have raised concerns about the implications that such effects might have for the patient's autonomous decision-making.^32^ These concerns are exacerbated in the psychiatric context, given the occasionally borderline capacity of patients with psychiatric disorders, the egosyntonic nature of some psychiatric conditions and the fact that the intended purpose of DBS in this context may be to alter the patient's dysfunctional emotional or motivational states.^7^

The MHA certification test might be suitable for NMD as a one-off, invasive intervention that is not associated with adverse psychosocial effects on the patient's self-conception or personality. However, it is not sufficiently sensitive to the potential impact of chronic DBS on the values that form the basis of a patient's decision-making. The concern about autonomy here may not pertain to the patient's understanding; rather, it may pertain to the way in which the intervention might affect the evaluative weight that the patient affords to the information at hand. In order to address this issue, we need to attend more closely to the evaluative weight that patients ascribe to this information in their decision-making process, and the intelligibility of these evaluations to the patient.

Notably, the Mental Capacity Act 2005 test of capacity includes the criterion that patients must be able to weigh and use material information as part of their decision-making process.^33^ Since the consent requirement for NMD in the MHA would be interpreted under the requirements of the Mental Capacity Act, this criterion is already implicitly incorporated in the MHA. There is long-standing debate concerning the appropriate role for capacity in the MHA that we cannot substantively engage with here.^34^ However, in the interests of transparent governance and because of the significance of the particular issues raised by DBS’ potential effects on the patient's values, the degree of risk it poses, and its highly experimental nature in this context, we believe that there is a particularly strong case for a more robust certification test for DBS. An appropriately robust test would require that patients must be certified as having capacity under the terms of the Mental Capacity Act.

The issue we are raising here is not whether MHA should or should not invoke considerations of capacity more generally; indeed, the MHA already stipulates that NMD is only lawful for a patient who has been certified as capable in some sense. Rather, the issue here is that the MHA explicitly employs a definition of capacity in this stipulation that ignores a key element of the concept as it is defined in the Mental Capacity Act. Crucially, it is this missing element, concerning the capacity to weigh and use material information that is often central to autonomy-based concerns with DBS.

One further way in which the consent safeguards for DBS could be bolstered is by employing an augmented diachronic consent procedures of the sort that some of us have outlined elsewhere.^35^^,^^36^ Augmented approaches may involve engaging with the patient about both their understanding of material information and their reasons for consenting to treatment at multiple stages, both before and during the course of treatment. In addition, augmented consent may involve seeking supplementary contributions from surrogate decision-makers, medical professionals and patient advocates. By allowing the clinician to develop a deeper multifaceted understanding of the patient's values over time, their role in their decision-making process, and how those values may be realised, or perhaps even themselves affected by treatment, such procedures would serve to enhance the power of the consent process as a facilitator and safeguard for patient autonomy.

It might seem counterintuitive to claim that DBS should be subject to a more stringent capacity test than NMD. After all, as we suggested above, the risk associated with DBS might be understood to be lower than those associated with NMD, because of the fact that DBS is largely reversible. Despite this, we believe that taking a more stringent approach to capacity in DBS is justified. First, and most importantly, NMD is a one-off, irreversible procedure, whereas DBS is a diachronic, ongoing and reversible medical intervention with multiple points at which consent may need to be solicited, and capacity assessed. This means that it is crucial that the assessments of capacity to consent to DBS are sensitive to the value changes that patients may undergo over the course of long-term treatment. Second, while adverse psychosocial effects on the patient's self-conception following DBS have been reported,^4^ there is less evidence associating NMD with such effects.^31^ Although there is presently little comparative evidence to allow us to make accurate likewise comparisons in this regard, if further evidence established that DBS does have stronger adverse effects on personality or self-conception than NMD, this would lend further support to the thought that considerations about how the patient is weighing the values at stake in their treatment decisions are particularly salient in DBS.

#### Recommendation 2

The MHA section governing DBS treatment should require that the patient is certified as having capacity under the Mental Capacity Act. In the context of DBS, such assessments may be aided by augmented diachronic consent procedures.

## Conclusion

The forthcoming revisions of the MHA must address the lack of provisions for DBS in psychiatry. However, we should not rectify this by treating DBS as legally equivalent to NMD. Instead, the MHA revisions should incorporate a set of DBS-specific provisions, much like the 2007 revisions introduced a specific section for ECT. These provisions should include a more robust certification test requiring that the patient is assessed as having capacity under the Mental Capacity Act. Furthermore, these provisions should only not apply for emergency treatment that is immediately necessary to save life or to prevent a serious deterioration of the patient's condition. Such provisions would (i) allow the MHA to avoid arbitrary inconsistency and rule out uses of DBS to which professional guidelines have objected and (ii) render the MHA more sensitive to the autonomy-related concerns raised by DBS in psychiatry. Furthermore, this strategy would enable greater flexibility to alter the regulation of DBS in the future, in the light of emerging evidence.
